# Taxonomic revision of Madagascan *Rhantus* (Coleoptera, Dytiscidae, Colymbetinae) with an emphasis on Manjakatompo as a conservation priority

**DOI:** 10.3897/zookeys.350.6127

**Published:** 2013-11-14

**Authors:** Anna Emilia Hjalmarsson, Rasa Bukontaite, Tolotra Ranarilalatiana, Jacquelin Herisahala Randriamihaja, Johannes Bergsten

**Affiliations:** 1 Department of Entomology, Swedish Museum of Natural History, Box 50007, SE-10405 Stockholm, Sweden; 2 Biodiversity and Climate Research Centre (BiK-F), Georg-Viogt-Straße 14-16, D-60325 Frankfurt am Main, Germany; 3 Department of Zoology, Stockholm University, SE-10405 Stockholm, Sweden; 4 Departement d’Entomologie, Faculté des Sciences, B.P. 906, Université d’Antananarivo, Antananarivo, Madagascar; 5 Programme National de Lutte contre le Paludisme de Madagascar, Androhibe, Antananarivo (101), Madagascar

**Keywords:** Diving beetles, Madagascar, GMYC, species delimitation, refugium, lectotype designation, new synonymy

## Abstract

We review the diving-beetle genus *Rhantus* Dejean of Madagascar (Coleoptera, Dytiscidae, Colymbetinae) based on museum collection holdings and recently collected expedition material. Both morphology and DNA is used to test species boundaries, in particular whether newly collected material from the Tsaratanana mountains in the north represent a new species or are conspecific with *Rhantus manjakatompo* Pederzani and Rocchi 2009, described based on a single male specimen from the central Ankaratra mountains. DNA of the holotype of *R. manjakatompo* was successfully extracted in a non-destructive way and sequenced. The general mixed Yule coalescent model applied to an ultrametric tree constructed from mitochondrial cytochrome c oxidase subunit I (*COI*) sequence data delimited three species. Morphological characters supported the same species unambiguously. We therefore recognise three species of *Rhantus* to occur in Madagascar: *R. latus* (Fairmaire, 1869), *R. bouvieri* Régimbart, 1900 and *R. manjakatompo* Pederzani and Rocchi, 2009. All three species are endemic to Madagascar and restricted to the highlands of the island. *Rhantusstenonychus* Régimbart, 1895, **syn. n.,** is considered a junior synonym of *R. latus*. We designate lectotypes for *R. bouvieri* and *R. goudoti* Sharp, 1882, the latter a junior synonym of *R. latus*. We provide descriptions, a determination key, SEM-images of fine pronotal and elytral structures, distribution maps, habitus photos, and illustrations of male genitalia and pro- and mesotarsal claws. We discuss the role of the Manjakatompo forest as a refugium for Madagascan *Rhantus* diversity and other endemics of the montane central high plateau.

## Introduction

*Rhantus* Dejean is a large cosmopolitan genus of medium-sized aquatic diving beetles. Several studies have pointed out that the genus as presently defined is clearly paraphyletic and will likely be split in the future (Balke et al. 2007). *Rhantus* has a track record of colonizing oceanic islands and forming island endemics (Balke et al. 2007, 2009), which indicates capacity for infrequent long-distance dispersal. It has colonized several islands in the Pacific with island-endemics on Fiji (Balke et al. 2007), New Caledonia (Balke et al. 2010), Galapagos (Peck and Balke 1993) and Hawaii (Balke 1993) to name a few. The Afrotropical region harbours only a modest part of the global *Rhantus* diversity (Guignot 1961). Balke (1992a) revised the *Rhantus* species of the Mascarene Islands outside of Madagascar and concluded that previous records were erroneous and that three species were endemic for this area, each to one of the three islands Mauritius, La Reunion and Ile Rodrigues. The Mascarene Islands are of rather recent volcanic origin (8-15 mya) and are known to have been on the receiving end of flora and fauna from Madagascar. Madagascar itself is a large island with ecosystems spanning from rainforests to semi deserts. The level of endemism is extremely high which has granted Madagascar a top placement among biodiversity hotspots (Myers et al. 2000). But the fauna is also known for its microendemic patterns – species distributions are often restricted to smaller geographical areas, almost like “islands within the island”. Some colonizations have led to rich allopatric species radiations, but the seemingly low species diversity of *Rhantus* perhaps indicates that their relatively high dispersal capacity has prevented the genus from a significant radiation.

The first *Rhantus* species described from Madagascar was *Rhantus latus* (Fairmaire 1869, in the genus *Colymbetes* Clairville). Since then four additional species have been described from the island, but the validity of some of these are questionable (Balke 1992b, Pederzani and Rocchi 2009) and no modern revision exist. In addition, apart from *Rhantus latus*, Madagascan *Rhantus* are very poorly represented in collections and are seemingly rare. Recent expeditions by the Swedish Museum of Natural History in collaboration with the Entomology department of the University of Antananarivo, have unearthed significant new material of Madagascan *Rhantus*, especially from Manjakatompo forestry station in the Ankaratra mountain massif. The new material both enables the assessment of intraspecific character variation and the extraction and sequencing of DNA data to test species limits. The purpose of this study is to revise the Madagascan *Rhantus* species based on this material, type material and other museum holdings, notably from the collections at the Museé National d’Historie Naturelle in Paris. In particular, the discovery in 2004 of what seemed to be a new undescribed *Rhantus* species from the Tsaratanana mountain massif in the north of Madagascar warranted a revisonary treatment of the group. In 2009 however, Pederzani and Rocchi (2009) described a new *Rhantus* species from the Ankaratra mountains in central Madagascar based on a single male specimen. The description showed both clear similarities but also some differences to the new species discovered in 2004 and it was hypothesized that DNA data and explicit species delimitation tests could aid in resolving this taxonomic question. Results of all these studies are summarized in the presented paper.

### Material and methods

All specimens examined in this study are registered in the NHRS collection objects database (interface via www.naturarv.se) but are deposited in the following collections and referred to by the abbreviations:

BMNH Museum of Natural History, London, Great Britain;

MNHN Muséum National d’Histoire Naturelle, Paris, France;

NMW Naturhistorisches Museum Wien, Austria;

NHRS Swedish Museum of Natural History, Stockholm, Sweden;

DEUA Departement d’Entomologie, Université d’Antananarivo, Antananarivo, Madagascar.

Measurements were taken on specimens in a horizontal position. The following measurements were taken: ML, maximum length from head to tip of elytra; MW, maximum width; LP, length of pronotum medially; WPB, pronotal width at base; LE, length of elytra from tip of scutellum to apex. The measurements were taken using an Olympus SZX12 stereomicroscope with an Infinity X camera and a calibrated ruler tool in the software DeltaPix Insight 2.0. Environmental scanning electron microscopy was done using a FEI/Philips XL30 FEG ESEM at the Institute for Surface Chemistry, Stockholm, Sweden. The images were generated at 350× magnification, with a gaseous secondary electron detector in low vacuum mode; the accelerating voltage of the electron beam was 17 kV. In opposite to a backscattered detector, a gaseous secondary electron detector depicts depressions of the surface brightly.

### Molecular data

DNA was extracted from legs of ethanol-preserved material collected after 2004 using Qiagen DNeasy 96 Tissue kit (see Table 1 for specimen information). For the dry-mounted holotype of *Rhantus manjakatompo*, (collected 2001) the QIAamp® DNA Micro kit was used, following the protocol for animal tissue with incubation at 56°C overnight. A single hindleg was carefully removed and after incubation re-glued to the body. Two fragments of the gene cytochrome c oxidase subunit I (*COI*) were sequenced for analysis. Primers used for amplification and sequencing were derived from several sources (Table 2).

**Table 1.** Data on specimens sequenced for COI and Genbank accession numbers. First accession number is for the 3’ end (patdyt-jerry) and second accession number is for the 5’ end (lco-hco) of COI.

Cat. ID	Species	Locality	Date	Collector	Mus.	GB Acc. No.
BMNH-743391	Rhantus bouvieri	Andringitra	9.V.2006	Bergsten et al	NHRS	KF548613, KF548639
BMNH-743392	Rhantus bouvieri	Andringitra	9.V.2006	Bergsten et al	BMNH	KF548614, KF548640
BMNH-829990	Rhantus latus	Ambalavao, 15km SW of			BMNH	KF548615, KF548641
NHRS-JLKB000000089	Rhantus latus	Ambohijanahary	19.XII.2009	Bergsten et al	NHRS	KF548616, KF548642
NHRS-JLKB000000090	Rhantus latus	Ambohijanahary	19.XII.2009	Bergsten et al	NHRS	KF548617, KF548643
BMNH-741961	Rhantus latus	Ambositra, 34km S of	06.V.2006	Bergsten et al	BMNH	KF548618, KF548644
BMNH-829991	Rhantus latus	Andasibe	04.I.2007	Isambert et al	BMNH	KF548619, KF548645
BMNH-742090	Rhantus latus	Andringitra	9.V.2006	Bergsten et al	BMNH	KF548620, KF548646
BMNH-729860	Rhantus latus	Col des Tapia	8.XII.2004	Balke et al	BMNH	KF548621, KF548647
BMNH-729861	Rhantus latus	Col des Tapia	8.XII.2004	Balke et al	BMNH	KF548622, KF548648
BMNH-729862	Rhantus latus	Col des Tapia	8.XII.2004	Balke et al	BMNH	KF548623, KF548649
BMNH-729863	Rhantus latus	Col des Tapia	8.XII.2004	Balke et al	BMNH	KF548624, KF548650
BMNH-729864	Rhantus latus	Col des Tapia	8.XII.2004	Balke et al	BMNH	KF548625, KF548651
BMNH-829992	Rhantus latus	Col des Tapia	8.XII.2004	Balke et al	BMNH	KF548626, KF548652
BMNH-741810	Rhantus latus	Isalo	11.V.2006	Bergsten et al	BMNH	KF548627, KF548653
BMNH-742359	Rhantus latus	Sendrisoa	7.V.2006	Bergsten et al	BMNH	KF548628, KF548654
BMNH-741979	Rhantus latus	Zombitse	14.V.2006	Bergsten et al	BMNH	KF548629, KF548655
BMNH-742639	Rhantus latus	Zombitse	15.V.2006	Bergsten et al	BMNH	KF548630, KF548656
BMNH-742640	Rhantus latus	Zombitse	15.V.2006	Bergsten et al	BMNH	KF548631, KF548657
BMNH-742641	Rhantus latus	Zombitse	15.V.2006	Bergsten et al	BMNH	KF548632, KF548658
BMNH-729851	Rhantus latus				BMNH	KF548633, KF548659
NHRS-JLKB000030412	Rhantus manjakatompo	Manjakatompo	8.X.2001	Gerecke & Goldschmidt	NMW	KF548634, na
BMNH-672725	Rhantus manjakatompo	Tsaratanana	20–24.XII.2004	Lees & Ranaivosolo	BMNH	KF548635, KF548660
BMNH-672726	Rhantus manjakatompo	Tsaratanana	20–24.XII.2004	Lees & Ranaivosolo	BMNH	KF548636, KF548661
BMNH-672730	Rhantus manjakatompo	Tsaratanana	20–24.XII.2004	Lees & Ranaivosolo	NHRS	KF548637, KF548662
BMNH-672731	Rhantus manjakatompo	Tsaratanana	20–24.XII.2004	Lees & Ranaivosolo	NHRS	KF548638, KF548663

**Table 2.** Primers used for the PCR to amplify two fragments of COI.

Primer	Direction	Sequence (5’-3’)
PatDyt^1^	Reverse	TCATTGCACTAATCTGCCATATTAG
Jerry^2^	Forward	CAACATTTATTTTGATTTTTTGG
LCO^3^	Forward	GGTCAACAAATCATAAAGATATTGG
HCO^3^	Reverse	TAAACTTCAGGGTGACCAAAAAATCA

^1^Isambert et al. (2011)

^2^Simon et al. (1994)

^3^Folmer et al. (1994)

DNA fragments were PCR amplified using “Ready-to-go” PCR Beads from Pharmacia Biotech and Phire Hot Start II PCR Master mix following the manufacture’s standard protocols. The thermal cycling profile for “Ready-to-go” PCR was 95°C for 5 min, followed by 40 cycles of 95°C for 30 s, 50°C for 30 s, 72°C for 1 min and finally 72°C for 8 min. PCR cycling for Phire Hot Start PCR was 98°C for initial denaturation 30 s, followed by 40 cycles of 98°C for 5 s, 53°C for 5 s, 72°C for 15 s, 72°C for 1 min. Product yield, specificity of amplification and contamination were investigated using agarose gel electrophoresis. PCR products were purified using ExoFAP Cleanup mix and cycle sequenced using the same primers used to amplify. For sequencing reactions the ABI BigDye Terminator kit ver. 3.1 was used. Sequencing products were purified using the DyeEx 96 kit and fragments were analysed on an ABI377xl analyser from Applied Biosystems. Gene regions were sequenced in both directions. The contigs were assembled from the forward and reverse reads and primers were trimmed in Sequencher 5.0 (Gene Codes Corporation 2011). All sequences are deposited in Genbank under the accession numbers KF548613 - KF548663 (Table 1).

### Analyses

Sequence data for 26 specimens were aligned in ClustalX 2.1 (Larkin et al. 2007) using default settings. The resulting combined matrix based on the two gene fragments was gap-free and had a total length of 1486 base pairs. An ultrametric tree was obtained from Bayesian MCMC analysis conducted in MrBayes 3.2 (Ronquist et al. 2012). We used a GTR+I+*Γ* model jointly for all codon positions but allowed each position a separate rate multiplier. Branch lengths were estimated under an autocorrelated relaxed clock model (TK02). Two separate MCMC analyses were run for 200000 generations with chains sampled every 100 generations, and a burn-in of 10% was chosen after visual examination of the –lnL plotted against generations and the convergence statistics provided by the program. A species delimitation test was conducted using the General Mixed Yule-Coalescent (GMYC) approach (Pons et al. 2006) and the *splits* package (Ezard et al. 2009), implemented in R 2.14.0 (R Development Core Team 2011). The single threshold method was used on the tree to find the maximum likelihood solution of the transition point between coalescent and interspecific branching patterns on the tree. Genetic distances were calculated under the Kimura-2-parameter model using Mega 5.0 (Tamura et al. 2011).

## Results

### GMYC species delimitation

**Figure 1.**
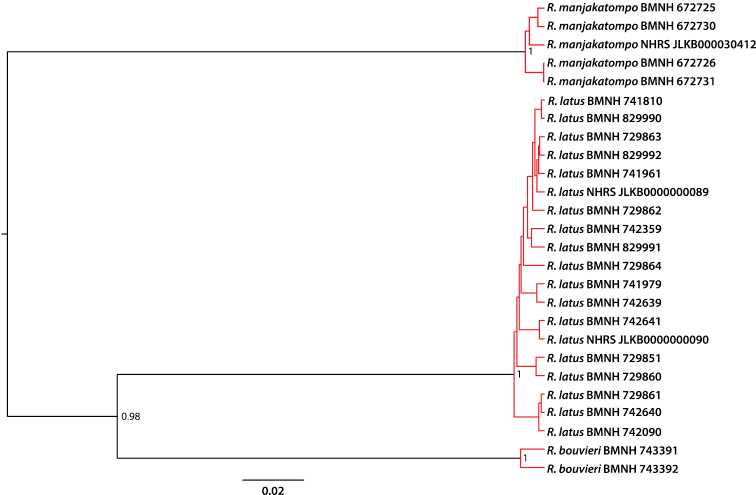
Ultrametric tree obtained from Bayesian analysis of of the two concatenated *COI* gene fragments. Red branches indicate separately coalescing clusters corresponding to species. Node values show posterior probability values and scale bar indicates the expected number of substitutions per site. “Rhantus manjakatompo NHRS–JLKB000030412” is the holotype of *Rhantus manjakatompo*.

The GMYC model was significantly better than a single coalescence model with a likelihood ratio test (lgL GMYC: 158.9, lgL single coalescence: 149.4, p = 0.0003), and divided the terminals into tree separately coalescing units: *Rhantus manjakatompo*, *Rhantus latus* and *Rhantus bouvieri* (Figure 1). No other solution had a log-likelihood value within 2 units (an approximate confidence interval) of the maximum likelihood solution. The holotype of *Rhantus manjakatompo* (NHRS-JLKB000030412) from Ankaratra mountains was nested within the single coalescing unit represented by the four specimens from the Tsaratanana mountains (Figure 1). The genetic distance between the holotype from Ankaratra and the specimens from Tsaratanana was 0.008-0.011, which was within the range among the Tsratanana specimens alone (0-0.012). The within-species genetic variation was 0.015 for *Rhantus bouvieri* and 0-0.019 (mean = 0.009) for *Rhantus latus*. The distances between the three species were almost an order of magnitude greater: 0.11-0.13 between *Rhantus bouvieri* and *Rhantus latus*, 0.17 between *Rhantus bouvieri* and *Rhantus manjakatompo* and 0.16-0.17 between *Rhantus latus* and *Rhantus manjakatompo*.

### Taxonomic part

#### Rhantus manjakatompo

Pederzani & Rocchi, 2009

http://species-id.net/wiki/Rhantus_manjakatompo

**Figure 2.**
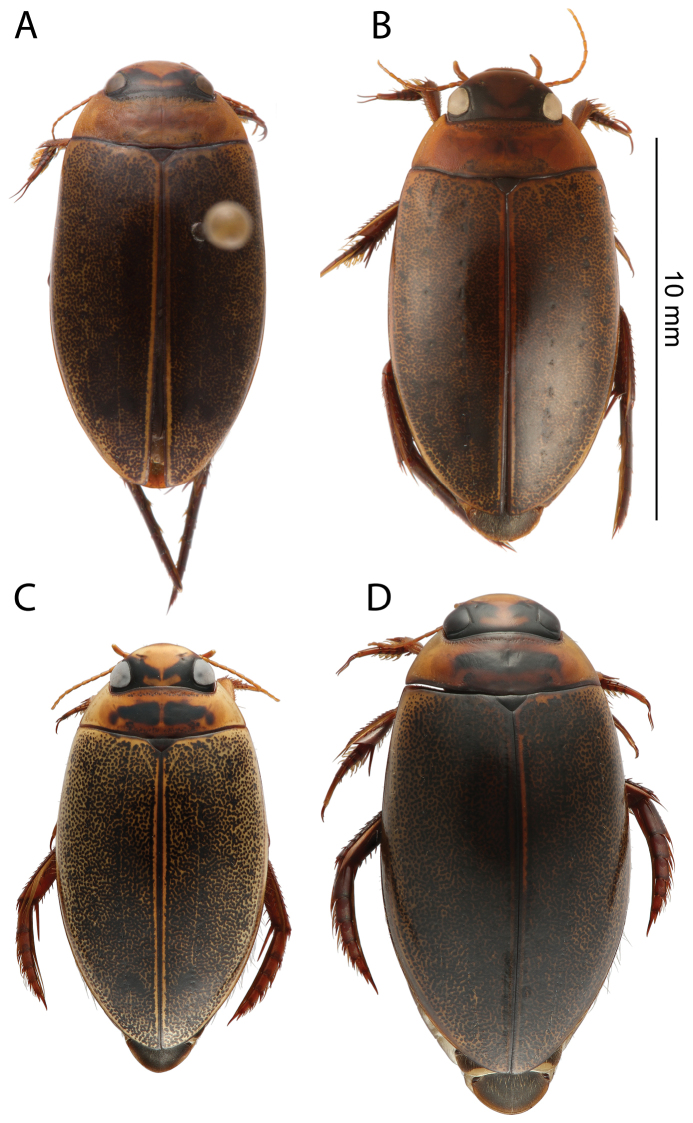
Habitus, dorsal view: **A** *Rhantus manjakatompo* holotype (Ankaratra mountains) **B** *Rhantus manjakatompo* (Tsaratanana mountains) **C** *Rhantus bouvieri* **D** *Rhantus latus*.

**Figure 3.**
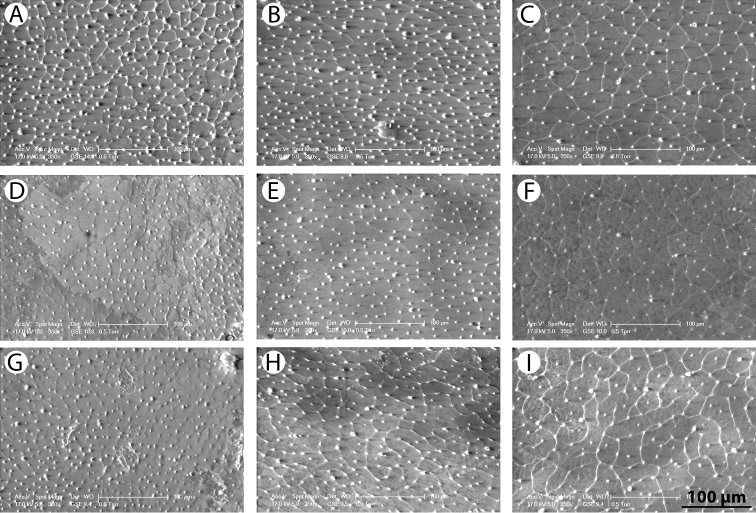
SEM images of pronotal and elytral microstructure: **A–C** *Rhantus manjakatompo* pronotum (**A**) anterior part of elytron (**B**) and posterior part of elytron (**C**) **D–F** *Rhantus bouvieri* pronotum (**D**) anterior part of elytron (**E**) and posterior part of elytron (**F**) **G–I** *Rhantus latus* pronotum (**G**) anterior part of elytron (**H**) and posterior part of elytron (**I**).

**Figure 4.**
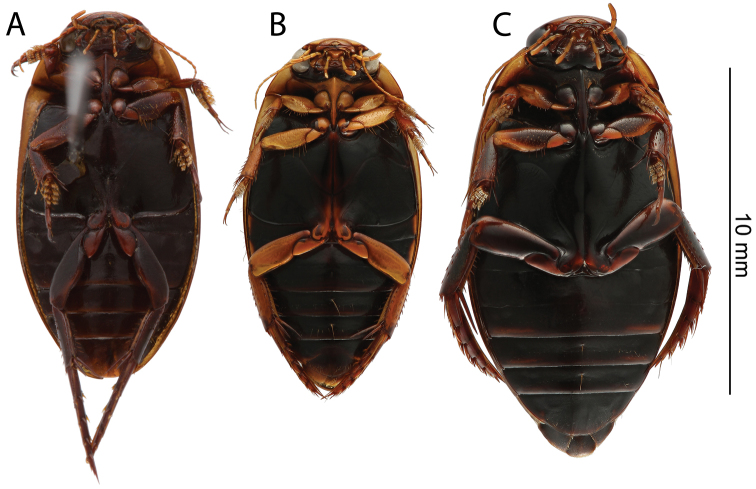
Habitus, ventral view: **A** *Rhantus manjakatompo* holotype **B** *Rhantus bouvieri* **C** *Rhantus latus*.

**Figure 5.**
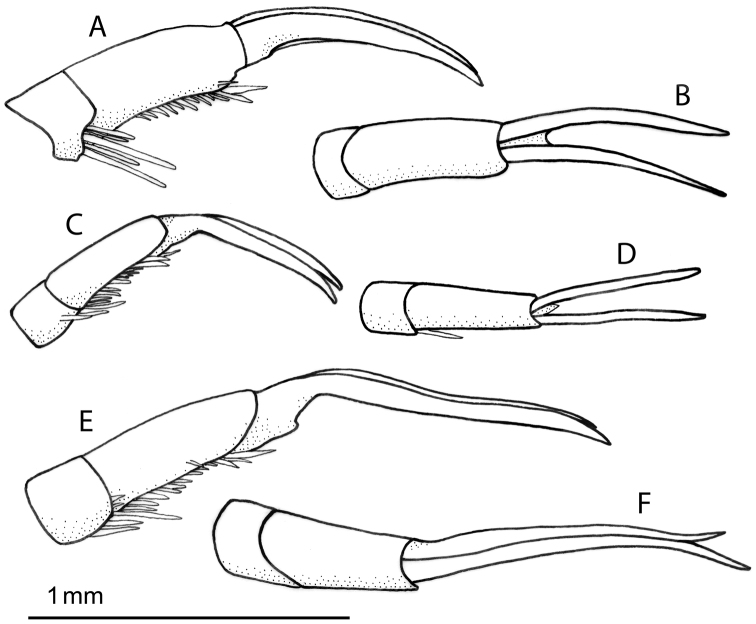
Left protarsal claws: **A–B** *Rhantus manjakatompo*,lateral (**A**) and dorsal (**B**) view **C–D** *Rhantus bouvieri*, lateral (**C**) and dorsal (**D**) view **E–F** *Rhantus latus*, lateral (**E**) and dorsal (**F**) view.

**Figure 6.**
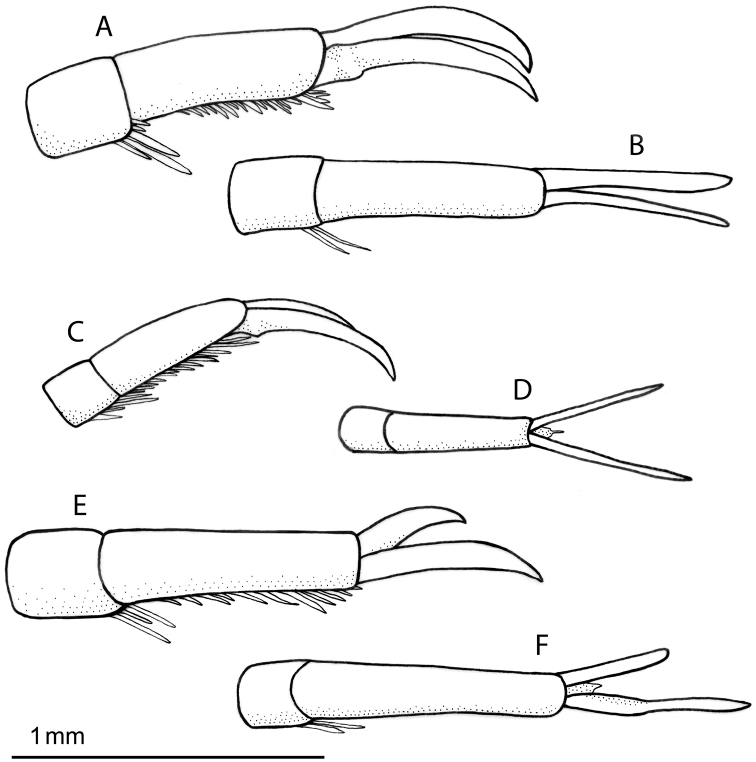
Left mesotarsal claws: **A–B** *Rhantus manjakatompo*,lateral (**A**) and dorsal (**B**) view **C–D** *Rhantus bouvieri*, lateral (**C**) and dorsal (**D**) view **E–F** *Rhantus latus*, lateral (**E**) and dorsal (**F**) view.

**Figure 7.**
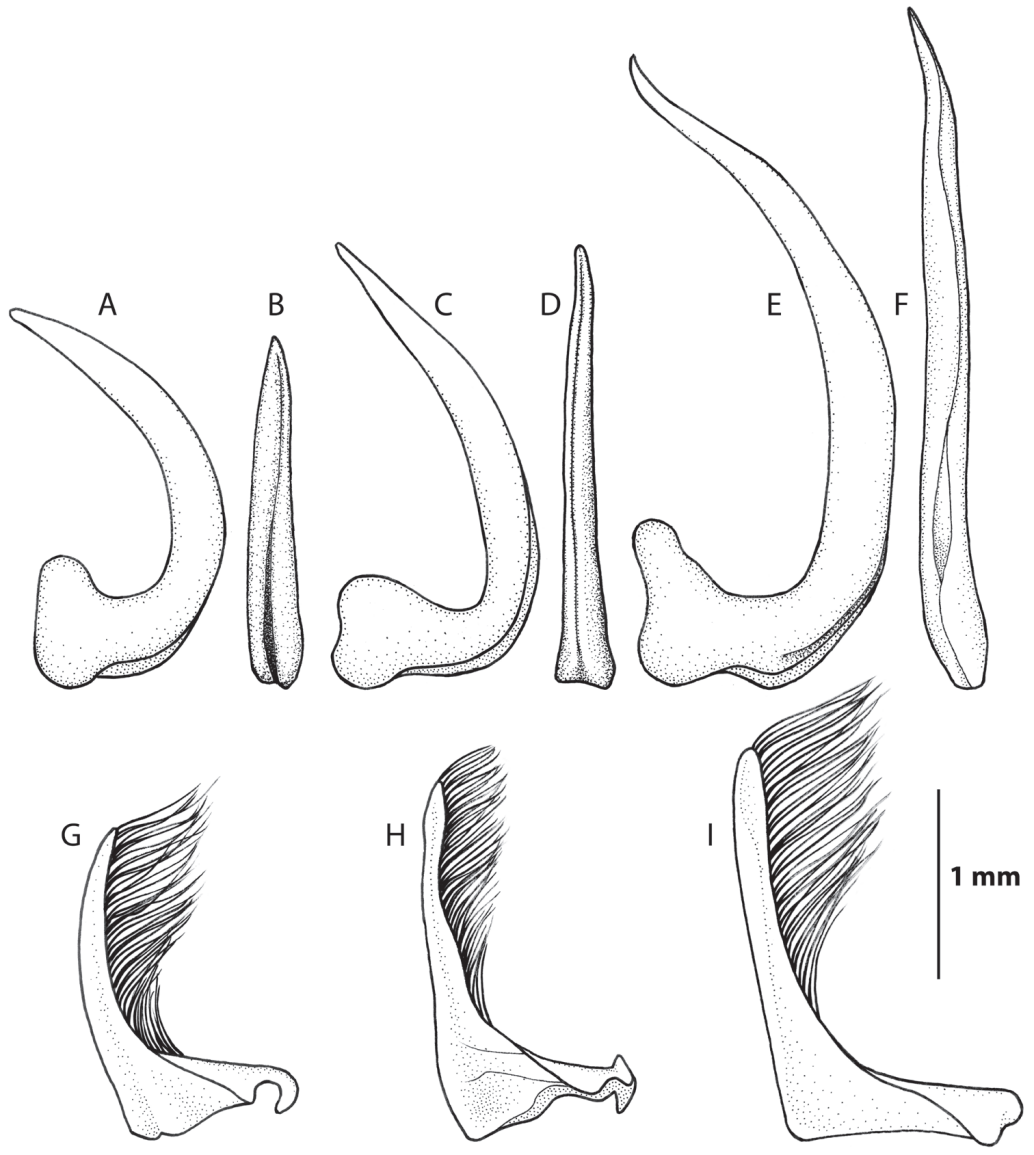
Male genitals, penis in lateral and dorsal view: **A–B** *Rhantus manjakatompo* **C–D** *Rhantus bouvieri* **E–F** *Rhantus latus*. Parameres in lateral view **G** *Rhantus manjakatompo* **H** *Rhantus bouvieri* **I** *Rhantus latus*.

Figures 2a–b , 3a–c , 4a , 5a–b , 6a–b , 7a–b, g

Rhantus manjakatompo Pederzani & Rocchi, 2009: 88

##### Type locality.

Madagascar, Antananarivo province, Ankaratra mountains, Manjakatompo reserve.

**Type material** (NMW). Holotype *♂* “Madagascar, Ankaratra (Antananarivo), Reserve Manjakatompo, spring stream exp. SE. N deviation to Analamitana (left affl. MD 107) m 1850 asl, 8.x.2001, 16.0°C, 0.003 mS/cm. Gerecke & Goldschmidt collectors BMNH (E) 2004-46”, “Holotype Rhantus manjakatompo Pederzani & Rocchi 2008”, “Data in NHRS-JLKB 000030412” “DNA Voucher”.

**Additional material studied** (NHRS, BMNH, DEUA, see Appendix): **Sofia region (former provinces: Mahajanga):** 2*♂*, 2*♀* (Cat. No. BMNH-672731, 672730, 672726, 672725): Tsaratanana [Antetykalambazaha Camp], 14.1824S, 48.9448E, 1700m, 20–24.xii.2004, Malaisetrap, Leg. Lees, Ranaivosolo.

**Vakinankaratra region (former provinces: Antananarivo):** 4spp. (Cat. No. NHRS-JLKB000021018) Manjakatompo [Analamitana] [swamp near stream][MJK12-02], 19.3640S, 47.2991E, 1760m, 22.i.2012, Leg. Ranarilalatiana, Randriamihaja, 1sp. (Cat. No. NHRS-JLKB000021019) Manjakatompo [Tavolatara][swamp near stream][MJK12-08], 19.3491S, 47.2784E, 2050m, 24.i.2012, Leg. Ranarilalatiana, Randriamihaja, 5spp. (Cat. No. NHRS-JLKB000021020) Manjakatompo [Tavolatara][pool near stream][MJK12-09], 19.3491S, 47.2780E, 2050m, 24.i.2012, Leg. Ranarilalatiana, Randriamihaja, 11spp. (Cat. No. NHRS-JLKB000021021) Manjakatompo [Tavolatara][swamp near source][MJK12-10], 19.3496S, 47.2779E, 2050m, 24.i.2012, Leg. Ranarilalatiana, Randriamihaja, 3spp. (Cat. No. NHRS-JLKB000021022) Manjakatompo [Anosiarivo][lake near source][MJK12–13], 19.3449S, 47.3041E, 2070m, 24.i.2012, Leg. Ranarilalatiana, Randriamihaja, 1spp. (Cat. No. NHRS-JLKB000021023) Manjakatompo [Andongolongo][pool near source][MJK12–12], 19.3536S, 47.3006E, 1900m, 24.i.2012, Leg. Ranarilalatiana, Randriamihaja.

##### Diagnosis.

Pronotum entirely testaceous or with two small dark spots medially (Figure 2a–b). Black irrorations of elytra somewhat confluent subapically (Figure 2a–b). Male protarsal claws equally long, evenly curved, apex acute (Figure 5a–b). Male mesotarsal claws equally long but posterior claw distinctly thicker than anterior claw (Figure 6a–b). Penis short and robust (Figure 7a–b), Parameres evenly curved and tapering to apex (Figure 7g). 11.5–12.5 mm long.

##### Description.

*Size*: ML 11.5–12.5 mm; MW 5.7–5.9 mm; Lp 1.4 mm; Epb 4.7–4.9 mm; Le 8.4–9.0 mm (n = 5).

*Head*. Testaceous with black areas posteriorly and inside eyes. Interocular black pattern narrowly separated medially (Figure 2a–b). Dense reticulation of well impressed meshes, very fine punctuation within and at edges of meshes.

*Pronotum*. Testaceous to ferrugineous with two small dark spots medially (Figure 2b), which may be absent (Figure 2a). Rim at lateral margin clearly visible to indistinct. Dense reticulation of well impressed meshes, very fine punctuation within and at edges of meshes (Figure 3a).

*Elytra*. Testaceous with black irrorations, leaving paler sides and yellow sutural lines (Figure 2a–b). Black irrorations somewhat confluent subapically. Reticulation of polygonal meshes simple anteriorly and double at middle and posteriorly, meshes are somewhat less impressed than on pronotum and larger posteriorly. Very fine punctuation within and at edges of meshes (Figure 3b–c).

*Ventral side*. Dark brown to black. Epiplura testaceous. Metafemora infuscated (Figure 4a).

*Male*. Protarsal claws equally long, evenly curved, apex acute (Figure 5a–b). Mesotarsal claws equally long but posterior claw distinctly thicker than anterior claw (Figure 6a–b). Penis short and robust, shape as Figure 7a–b. Parameres evenly curved and tapering to apex (Figure 7g).

##### Remarks.

In 2004 David Lees and Ravomiarana Ranaivosolo collected material of what seemed to be a new *Rhantus* species from the mountain massif of Tsaratanana, North Madagascar. *Rhantus manjakatompo* was described in 2009 based on a single male specimen from Ankaratra mountains, 70km south of Antananarivo in central Madagascar. Despite variation in color and impression of a lateral rim of pronotum, molecular data indicate that the studied material from Ankaratra and Tsaratanana are conspecific or at least not an old enough divergence to be recognised based on *COI* sequence (genetic distance 0.008–0.011). As also male tarsal claws, aedeagus and parameres were identical, we consider the specimens from Tsaratanana mountains conspecific with the holotype of *Rhantus manjakatompo*.

##### Habitat.

Associated with sources and streams, and surrounding water assemblages like nearby pools and marshes at altitudes between 1700 to 2070 m a.s.l. In Manjakatompo, the species was most numerous at elevations above 2000 m.

##### Distribution.

**Figure 8.**
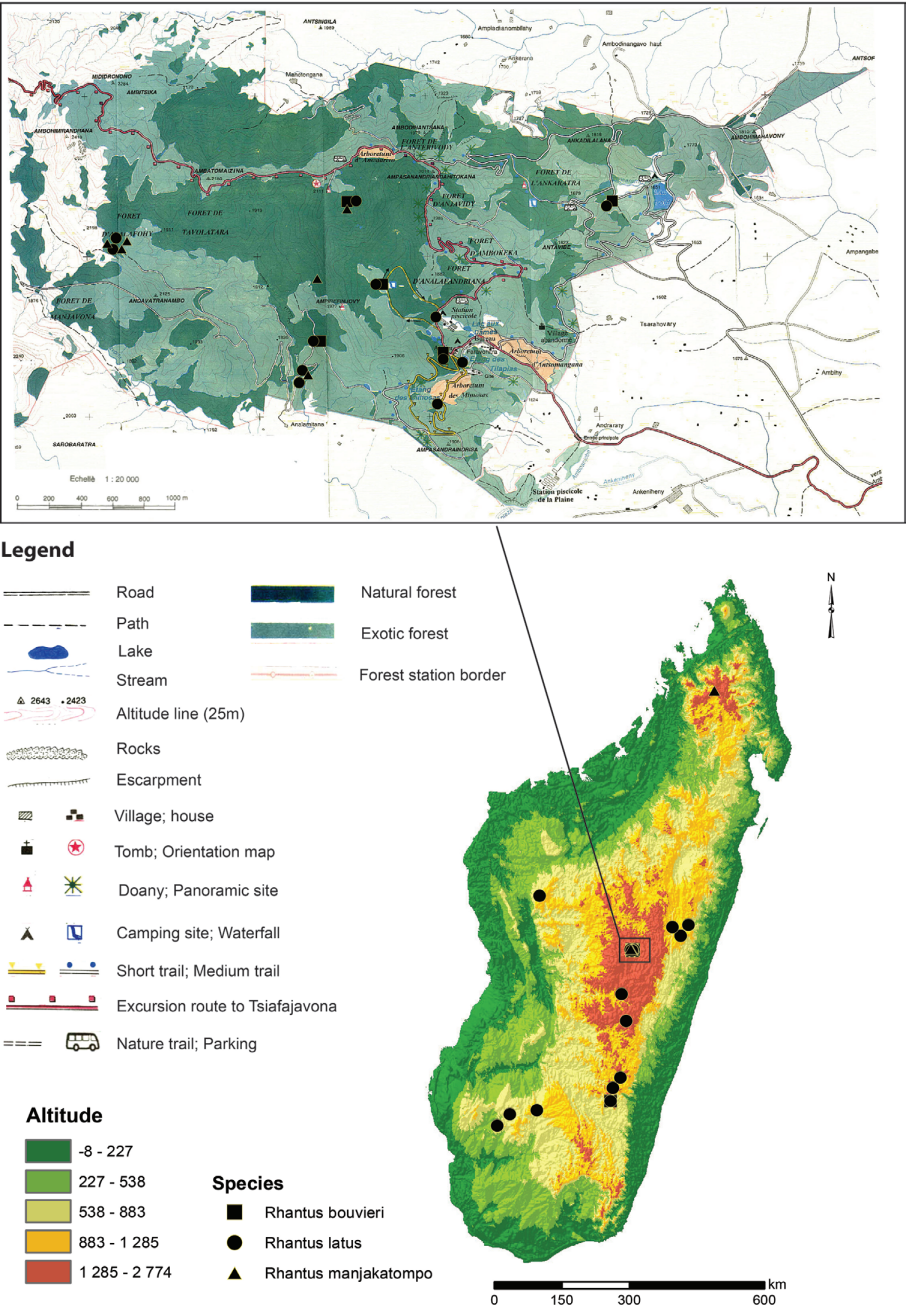
Distribution and all known records of the three *Rhantus* species of Madagascar with a special emphasis on Manjakatompo. Inset map of Manjakatompo adapted from FTM (1995) Extent of natural forest left may have changed since the map was made.

Endemic to Madagascar. Only known from Tsaratanana mountains and Manjakatompo forestry station in the Ankaratra mountains (Figure 8).

#### Rhantus bouvieri

Régimbart, 1900

http://species-id.net/wiki/Rhantus_bouvieri

Figures 2c , 3d–f , 4b , 5c–d , 6c–d , 7c–d, h

Rhantus bouvieri Régimbart, 1900: 374
Rhantus Bouvieri Régimbart, 1899: Zimmerman (1920: 199); Guignot (1961: 754).
Rhantus bouvieri Régimbart, 1899: Nilsson (2001: 48).

##### Type locality.

Madagascar, Fianarantsoa.

##### Type material

(MNHN). **Lectotype** *♂*, here designated to fix the concept of *Rhantus bouvieri* and to ensure the universal and consistent interpretation of the same: “Data in NHRS-JLKB000030144”, “Rhantus Bouvieri Rég. M. Régimbart det.”, “Museum Paris Madagascar Fianarantsoa Grandidier 1852–91”, ”Rhantus bouvieri Régimbart, 1900 Det. J. Bergsten 2011” our lectotype label.

**Paralectotypes** 2*♀*: “Data in NHRS-JLKB000030405 and Data in NHRS-JLKB0000304283”, “Museum Paris Madagascar Fianarantsoa Grandidier 1852–91”, our paralectotype labels.

**Additional material studied** (NHRS, BMNH, MNHN, NMW, DEAU, see Appendix):

**Matsiatra Ambony (Haute Matsiatra) region (former provinces: Fianarantsoa):** 2*♂* (Cat. No. BMNH-743391, 743392), Andringitra NP [Zomandao river, by bridge on road to camp Belamba], 22.1043S, 46.9207E, 1420m, 09.v.2006, Bottletrap, Leg Bergsten et al.

**Vakinankaratra region (former provinces: Antananarivo):** 1*♂*, 1*♀* (Cat. No. NHRS-JLKB000030410, 30411), Manjakatompo [pond by], 10.x.1968, Leg. Starmühlner, 2spp. (Cat. No. NHRS-JLKB000010272), Manjakatompo [Analafandriana, 500m S fish farm by the road][grassy pond][MAD11–14], 19.3619S, 47.3150E, 1730m, 03.xi.2011, Leg. Bergsten, Ranarilalatiana, Randriamihaja, Bukontaite, 8spp. (Cat. No. NHRS-JLKB000010275), Manjakatompo [500m E Lac Froid by the road][pond and inlet stream][MAD11–16] 19.3449S, 47.3338E, 1620m, 04.xi.2011, Leg. Bergsten, Ranarilalatiana, Randriamihaja, Bukontaite, 16spp. (Cat. No. NHRS-JLKB000010276), Manjakatompo [500m E Lac Froid by the road][pond and inlet stream][MAD11-16], 19.3449S, 47.3338E, 1620m, 04.xi.2011, Leg. Bergsten, Ranarilalatiana, Randriamihaja, Bukontaite, 10spp. (Cat. No. NHRS-JLKB000010273), Manjakatompo [2km NE Amparandraindrisa][pond and stream][MAD11-18], 19.3607S, 47.3009E, 1770m, 05.xi.2011, Leg. Bergsten, Ranarilalatiana, Randriamihaja, Bukontaite, 4spp. (Cat. No. NHRS-JLKB000021024), Manjakatompo [Anosiarivo][lake near source][MJK12-13], 19.3449S, 47.3041E, 2070m, 24.i.2012, Leg. Ranarilalatiana, Randriamihaja, 7spp. (Cat. No. NHRS-JLKB000021025), Manjakatompo [Poste][lake with grass][MJK12-14], 19.3542S, 47.3081E, 1800m, 24.i.2012, Leg. Ranarilalatiana, Randriamihaja.

**Madagascar (region indecisive):** 3*♂*, 6*♀* (Cat. No. NHRS-JLKB000030139, 30140, 30141, 30142, 30144, 30400, 30401, 30402, 30403), Madagascar, Leg. Catat, 2*♀* (Cat. No. NHRS-JLKB000030404, 30143) Madagascar [Centre-Sud], 1901, Leg. Alluaud, 1*♀* (Cat. No.: NHRS-JLKB000030138) Antananarivo [city or province, indecisive], Leg. Sikora, 1*♀* (Cat. No. NHRS-JLKB000030287) Forêt Tanala [province indecisive], Leg. Alluaud

##### Diagnosis.

Pronotum with two elongated rectangular dark spots, narrowly (or partly) separated in middle (Figure 2c). Male protarsal claws equally long, straight in lateral view apart from at base and apex (Figure 5c–d). Male mesotarsal claws curved in lateral view, equally thin, the anterior claw somewhat longer than the posterior (Figure 6c–d). Penis in dorsal view evenly tapering towards apex, in lateral view with a relatively sharp angle at the base (Figure 7c–d). Parameres with inner margin undulated (Figure 7h). 9.4–10.8 mm long.

##### Description.

*Size*: ML, 9.4-10.8 mm;  MW, 5.2–5.7 mm; Lp, 1.0–1.4 mm; Wp, 3.8–4.1 mm; Le, 7.2–8.1 mm (n = 18).

*Head*. Testaceous with black areas inside and posterior of eyes. Interocular black pattern tapering towards the interior and narrowly separated (Figure 2c). Dense micropunctation and incomplete microreticulation.

*Pronotum*. Testaceous ferrugineous with two elongated rectangular black areas that are narrowly separated medially (Figure 2c). Lateral rim present, distinct. Dense micropunctation and incomplete microreticulation (Figure 3d).

*Elytra*. Testaceous with black irrorations, leaving paler sides and yellow sutural lines (Figure 2c). Somewhat confluent to form two small black areas subapically. Microreticulation double but somewhat indistinct. Very fine punctuation within and at edges of meshes (Figure 3e–f).

*Ventral side*. Dark brown to black, abdominal segments with testaceous spots along the lateral edges. Epiplura and legs, including metafemur, testaceous (Figure 4b).

*Male*. Protarsal claws equally long, medially straight in lateral view (Figure 5c-d). Mesotarsal claws curved in lateral view, equally thin, the anterior claw somewhat longer than the posterior (Figure 6c–d). Penis in dorsal view evenly tapering towards apex (Figure 7d). in lateral view not evenly curved but almost angulate at base (Figure 7c). Parameres with inner margin undulating (Figure 7h).

##### Remarks.

In the original description, Régimbart refers to specimens from Fianarantsoa collected by Grandidier, but the holotype is not designated. The three specimens labelled “Fianarantsoa Grandidier” at MNHN are therefore considered to be syntypes and a male with genitalia extracted is designated here as the lectotype. Lectotype and paralectotypes were labelled as such.

##### Habitat.

Known from a river in Andringitra and streams and grassy ponds in Manjakatompo, in both cases at altitudes between 1420 to 2070 m a.s.l.

##### Distribution.

Endemic to Madagascar. Precise localities only known from Manjakatompo forestry station in the Ankaratra mountains and the Andringitra mountains (Figure 8). Forêt Tanala, Fianarantsoa and Tananarive are less precise region descriptions which includes Manjakatompo (Antananarivo) and Andingitra (Fianarantsoa, Forêt Tanala).

#### Rhantus latus

(Fairmaire, 1869)

http://species-id.net/wiki/Rhantus_latus

Figures 2d , 3g–i , 4c , 5e–f , 6e–f , 7e–f, i

Colymbetes latus Fairmaire, 1869: 191. Type locality: Madagascar
Rhantus goudoti Sharp, 1882: 623. Synonymized by Branden (1885: 91). Type locality: Madagascar
Rhantus stenonychus Régimbart 1895: 179 **syn. n.** Type locality: Madagascar, lake Ambodinandohalo
Rhantus latus (Fairmaire, 1869): Régimbart (1895: 183); Zimmermann (1920: 202); Guignot (1961: 764); Nilsson (2001: 51).

##### Type material studied.

Typematerial of *Colymbetes latus* could not be localized in the Paris collections. We have no reasons to believe the type material is lost or destroyed, but to localize and identify the material that Fairmaire studied proved difficult as it was not found in Fairmaire’s collection. The type material was collected by medicine doctor and entomologist Charles Coquerel who died in 1867.

*Rhantus stenonychus*: **Holotype** *♂* for *Rhantus stenonychus* (MNHN): “Madagascar, Lac Ambodo, R.P. Camboue”, “Data in NHRS-JLKB000030296”.

*Rhantus goudoti*: **Lectotype** *♂* for *Rhantus goudoti*, here designated to fix the concept of Rhantus goudoti Sharp and to ensure the universal and consistent interpretation of the same (BMNH): “905”, “C. latus Fairm. seems very close to this, but is a little shorter in form, +paler” [handwritten note on the specimen], “Sharp Coll. 1905–313.”, “Type”, “Type 905 Goudoti Dej. Madagascar.” “Data in NHRS-JLKB 000030415“, our lectotype label. **Paralectotype** *♀* (BMNH) “Sharp Coll. 1905–313.”, “Co-type”, “Madagascar. 905” “Data in NHRS-JLKB 000030413“, our paralectotype label. **Paralectotype** *♂* (BMNH) “37” “Co-type”, “Madagascar 905”, “Sharp Coll. 1905–313.” “Data in NHRS-JLKB 000030414“our paralectotype label. 1*♂* **Paralectotype** (MNHN): Data in NHRS-JLKB 000030297, Colymbetes goudotii mihi Madagascar, D. Sharp Monogr., Ex Musæo Dejean, our paralectotype label.

**Additional material studied** (for full details see Appendix).

**Vakinankaratra region (former provinces: Antananarivo):** 15spp. (Cat No. NHRS-JLKB000010277), Manjakatompo [2km NE Amparandraindrisa][pond and stream][MAD11-18], 19.3607S, 47.3009E, 1770m, 05.xi.2011, Leg. Bergsten, Ranarilalatiana, Randriamihaja, Bukontaite, 24 spp. (Cat. No. NHRS-JLKB000010278), Manjakatompo [500m E Lac Froid by the road][pond and inlet stream][MAD11-16], 19.3449S, 47.3338E, 1620m, 04.xi.2011, Leg. Bergsten, Ranarilalatiana, Randriamihaja, Bukontaite, 12 spp. (Cat. No. NHRS-JLKB000010274), Manjakatompo [Analafandriana, 500m S fish farm by the road][grassy pond][MAD11-14], 19.3619S, 47.3150E, 1730m, 03.xi.2011, Leg. Bergsten, Ranarilalatiana, Randriamihaja, Bukontaite, 1sp. (Cat. No. NHRS-JLKB000010269), Manjakatompo [Analamitana, by bridge close to SKOL facility][stream and stagnant pool][MAD11-19], 19.3646S, 47.2989E, 1750m, 05.xi.2011, Leg. Bergsten, Ranarilalatiana, Randriamihaja, Bukontaite, 2sp. (NHRS-JLKB000010270), Manjakatompo [Analafandriana close to fish farm][stream and wet field][MAD11-13], 19.3581S, 47.3140E, 1730m, 03.xi.2011, Leg. Bergsten, Ranarilalatiana, Randriamihaja, Bukontaite, 1sp. (Cat. No. NHRS-JLKB000021026), Manjakatompo [Tavolatara][pool near stream][MJK12-09], 19.3491S, 47.2780E, 2050m, 24.i.2012, Leg. Ranarilalatiana, Randriamihaja, 2spp. (Cat. No. NHRS-JLKB000021027), Manjakatompo [Analamitana] [swamp near stream][MJK12-02], 19.3640S, 47.2991E, 1760m, 22.i.2012, Leg. Ranarilalatiana, Randriamihaja, 2spp. (Cat. No. NHRS-JLKB000021028), Manjakatompo [Tavolatara][swamp near source][MJK12-10], 19.3496S, 47.2779E, 2050m, 24.i.2012, Leg. Ranarilalatiana, Randriamihaja, 3spp. (cat. No. NHRS-JLKB000021029), Manjakatompo [Anosiarivo][lake near source][MJK12-13], 19.3449S, 47.3041E, 2070m, 24.i.2012, Leg. Ranarilalatiana, Randriamihaja, 2spp. (Cat. No. NHRS-JLKB000021030), Manjakatompo [Poste][lake with grass][MJK12-14], 19.3542S, 47.3081E, 1800m, 24.i.2012, Leg. Ranarilalatiana, Randriamihaja, 5sp. (Cat. No. NHRS-JLKB000021031), Manjakatompo [Andohariana][small lake][MJK12-07], 19.3677S, 47.3143E, 1710m, 24.i.2012, Leg. Ranarilalatiana, Randriamihaja, 10spp. (Cat. No. NHRS-JLKB000021032), Manjakatompo [near camping][big temp. lake][MJK12-15], 19.3630S, 47.3171E, 1710m, 25.i.2012, Leg. Ranarilalatiana, Randriamihaja.

**Alaotra Mangoro region (former provinces: Toamasina):** 1*♂* (Cat. No. NHRS-JLKB000030407), Mantadia, NP, Rianila Basin, [affluent non nommé riv.], [PO670], 18.935S, 48.4167E, 920m, 29.xi.1996, Leg. Legrand, Randriamasimanana, 1sp. (Cat. No. NHRS-JLKB000010268), Mantadia NP [3km from park entrance][open pond with vegetation][MAD11-42], 18.8526S, 48.4272E, 920m, 13.xi.2011, Leg. Bergsten, Ranarilalatiana, Randriamihaja, Bukontaite, 1*♀* (Cat. No. BMNH-829991), Andasibe NP [entry to park, Anamalozaotra river and pond][P61Bi01], 18.9348S, 48.4175E, 950m, 04.i.2007, Leg. Isambert et al., 2*♂* (Cat. No. NHRS-JLKB000030295), Antsianaka, 1892, Leg. Perrot, Perrot, 1*♂* (Cat. No. NHRS-JLKB000030289), Ambatosoratra, env. Tananarive, vii.1934, Leg. Olsoufieff.

**Matsiatra Ambony (Haute Matsiatra) region (former provinces: Fianarantsoa):** 1sp. (Cat. No. BMNH-742359), Sendrisoa, S of Ambalavao, [P38], 22.0098S, 46.9504E, 1160m, 07.v.2006, Leg. Bergsten et al., 1sp. (Cat. No. BMNH-742090), Andringitra NP [Zomandao river, by bridge on road to camp Belamba], 22.1043S, 46.9207E, 1420m, 09.v.2006, Leg. Bergsten et al., 1*♂* (Cat. No. BMNH-829990), Ambalavao, 15km SW of, RN7.

**Amoron’i Mania region (former provinces: Fianarantsoa):** 6spp. (Cat. No. BMNH-729860, 729861, 729862, 729863, 729864, 829992), Col des Tapia, 48 km N Ambositra, RN7, [P30MD33], 20.2388S, 47.1002E, 1440m, 08.xii.2004, Leg. Balke et al., 1*♂* (Cat. No. BMNH-741961), Ambositra, 34km S of, RN7, 20.7719S, 47.1810E, 1690m, 06.v.2006, Leg. Bergsten et al.

**Melaky region (Former provinces: Mahajanga):** 1*♂*, 1*♀* (Cat. No. NHRS-JLKB000000089, 000000090), Ambohijanahary NP [MAD09-76], 18.2685S, 45.4635E, 910m, 19.xii.2009, Leg. Bergsten, Ranarilalatiana, Randriamihaja, Jönsson

**Atsimo Andrefana region (former provinces: Toliara):** 3spp. (Cat. No. BMNH-742641, 742639, 742640), Zombitse-Vohibasia NP [Isoky, Ranomena, muddy pool in river basin], 22.6401S, 44.8644E, 580m, 15.v.2006, Leg. Bergsten et al., 1sp. (Cat. No. BMNH-741979), Zombitse-Vohibasia NP [edge of, Ambiamena, stagnant zebu-visited marshland, muddy & vegetation], 22.8601S, 44.6173E, 530m, 14.v.2006, Leg. Bergsten et al.

**Ihorombe region (former provinces: Fianarantsoa):** 1sp. (cat. No. BMNH-741810), Isalo NP [Menamaty river][river with vegetation][P41AM01], 22.5500S, 45.4012E, 760m, 11.v.2006, Leg. Bergsten et al.

**Analamanga region (former provinces: Antananarivo):** 1*♂* (Cat. No. NHRS-JLKB000030282), Antananarivo [city or province, indecisive], 31.v.1947, Leg. Clement, 1*♂* (Catl. No. NHRS-JLKB000030288), Lac Tsimbazaza, Antananarivo, 18.9333S, 47.5333E, 1410m, vii.1934, Leg. Vadon, 1*♂* (Cat. No. NHRS-JLKB000030284), Andrang, Leg. Sikora, 1*♂* (Cat. No. NHRS-JLKB000030285), Ambohibeloma, Leg. Sikora.

**Madagascar (region indecisive):** 2*♂*, 1*♀* (Cat. No. NHRS-JLKB000030406, 30408, 30409), Madagascar, vii.1968 – ix.1968, Leg. Starmühlner, 1sp. (Cat. No. BMNH-729851), Madagascar, 1*♂* (Cat. No. NHRS-JLKB000030286), Madagascar [Centre-Sud], Leg. Alluaud.

##### Diagnosis.

Pronotum somewhat infuscated and with one wide black spot, not divided medially (Figure 2d). Maleprotarsal claws long and slender, almost twice the length of last tarsal segment, somewhat sinuated and unequal, anterior claw longer (Figure 5e–f). Male mesotarsal claws very unequal, the anterior almost twice the length, and very broadened, compared to posterior (Figure 6e–f). Penis long and slender, apically upturning in lateral view, in dorsal view with the apex curved to the left and sharply pointed (Figure 7e–f). Parameres with dorsal edge straight (Figure 7i). 11.6–13.0 mm long.

##### Description.

*Size*:ML, 11.6–13.0 mm; MW, 6.2–7.0 mm; Lp,1.2–1.6 mm; Wp, 4.7–5.3 mm; Le, 9.3–10.1 mm (n = 12).

*Head*. Testaceous with black areas inside and posterior of eyes. Interocular black pattern often rather broadly separated medially (Figure 2d). Dense micropunctuation but no reticulation.

*Pronotum*. Somewhat infuscated and with one wide dark spot which is not medially divided (Figure 2d). No rim at lateral margin. Micropunctation and vague microreticulation (Figure 3g).

*Elytra*. Testaceous ferrugineous with black irrorations, leaving paler sides and yellow sutural lines (Figure 2d). Black irrorations regular throughout, even posteriorly with no sign of preapical black areas. Microreticulation double, meshes are well impressed posteriorly but vague anteriorly. Micropunctation within and at edges of meshes (Figure 3h–i).

*Ventral side*. Dark brown to black. Epiplura testaceous. Metafemur infuscated (Figure 4c).

*Male*. Protarsal claws long and slender, almost twice the length of last tarsal segment, somewhat sinuated and unequal, anterior claw longer (Figure 5e–f). Mesotarsal claws curved and unequal, the anterior claw is almost twice as long as the posterior and much broader (Figure 6e–f). Penis long and slender, apically upturning in lateral view, in dorsal view with the apex curved to the left and sharply pointed (Figure 7e–f). Parameres with dorsal edge straight (Figure 7i).

##### Remarks.

The interpretation of Fairmaire’s name *Colymbetes latus* is unambiguous following common usage (e.g. Régimbart 1895, Guignot 1961), even though typematerial with Coquerel as collector could not be found in Fairmaire’s collection in Paris, or elsewhere. Neither Guignot (1961) nor Balke (1992b) were able to localize the holotype of *Rhantus stenonychus* and the validity of the name, based on a single specimen, has therefore not been evaluated before but the name lingered in the literature. The details for the type in Régimbart’s original description are as follows: “Madagascar: Lac Ambodinandohalo (R. P. Camboué), un seul male (coll. R. Oberthür)”. In Régimbart’s collection at MNHN there was a pin without a specimen but with the handwritten labels by Régimbart “stenonychus Rég.” and “Madag. Coll. Oberthür”. We interpret the pin with the label as a reference to the Oberthür collection for the unique type. In Oberthür’s collection a single male specimen was found bearing the label “Lac Ambodo [or Ambod°], R.P. Camboue”. We believe “Lac Ambodo [or Ambod°],” is an abbreviation for Lac Ambodinandohalo and that this is the holotype. We are not able to find any records of another lake named “Ambodo” in Madagascar. Lake Ambodinandohalo was a lake in the haute-ville in Antananarivo and the French Jesuit priest and missionary Paul Camboué lived just west of Antananarivo in Arivonimamo and Ambohibeloma. *Peltodytes quadratus* Régimbart, 1895 was described in the same article from the same locality (Lac Ambodinandohalo, collected by R. P. Camboué) and the type specimen in Paris bore a label with the same abbreviated locality name (see Vondel 2010; Vondel and Bergsten 2012).

The holotype is as judged by morphological characters conspecific with *Rhantus latus*. Already in the original description similarities with *Rhantus latus* are obvious (also see Guignot 1961) and it is odd that Régimbart did not compare the species with *Rhantus latus* in his original description. We therefore synonymize the name *Rhantus stenonychus* with *Rhantus latus*.

##### Habitat.

Occurs in a quite wide range of habitats, like streams and rivers, muddy waterpools and grassy ponds. Of the three *Rhantus* species it is the only one that can be found below 1000 m altitude and known range include 530 to 2070 m.

##### Distribution.

Possibly endemic to Madagascar as we have not been able to verify the records from mainland Africa by Régimbart (1895) “Cafrerie, Cap” and by Guignot (1961) “Sud d’Afrique”. In Madagascar rather widespread over the central high plateau (Figure 8). Known from Andasibe-Mantadia NP, Antananarivo and Ambohijanahary NP, which are the northernmost records. Further known from several localities south of Antananarivo along RN7 from Manjakatompo forestry station to Andringitra NP, and further southwest to Isalo NP and Zombitse-Vohibasia NP. Seemingly lacking from the northern third of Madagascar.

### Identification key on habitus of females and males

**Table:** 

1a	Smaller (ML 9.4–10.8 mm), legs including metafemur mostly yellow (Figure 4b), pronotum yellow with two elongated rectangular black fields narrowly divided in middle (Figure 2c)	*Rhantus bouvieri*
1b	Larger (ML: 11.5–13.0 mm), legs mostly infuscated especially metafemur (Figure 4a, c), pronotum yellow or infuscated with or without black markings which, if present, are either not medially divided or are not elongated but small dots (Figure 2a–b, d).	2
2a	Pronotum entirely yellow without black markings (Figure 2a) or with two small black dots, medially divided (Figure 2b). Elytral black irrorations somewhat confluent subapically to form small dark fields (Figure 2a–b)	*Rhantus manjakatompo*
2b	Pronotum somewhat infuscated and with a single medial elongated rectangular black field not medially divided (Figure 2d). Black elytral irrorations even throughout, not forming denser black areas subapically (Figure 2d)	*Rhantus latus*

### Identification key for males

**Table:** 

1a	Male mesotarsal claws very unequal in length, anterior claw broad and almost twice as long as posterior claw (Figure 6e–f). Male protarsal claws very long, slender and sinuate, almost twice the length of last protarsal segment (Figure 5e–f). Penis long and slender, apically twisted (Figure 7e–f)	*Rhantus latus*
1b	Male mesotarsal claws equal or somewhat unequal, anterior claw thin (Figure 6a–d). Male protarsal claws shorter and not sinuated (Figure 5a–d). Penis shorter and not apically twisted (Figure 7a–d)	2
2a	Smaller (ML 9.4–10.8 mm), Male mesotarsal claws subequal, posterior claw somewhat shorter but equally thin as anterior claw (Figure 6c–d). Pronotum with two wide rectangular dark spots (Figure 2c). Penis in lateral view not evenly curved but almost angulate at base (Figure 7c). Parameres with inner margin undulating (Figure 7h)	*Rhantus bouvieri*
2b	Larger (ML: 11.5–12.5 mm), Male mesotarsal claws equally long but posterior claw distinctly thicker than anterior claw (Figure 6a–b). Pronotum with two small dark spots (Figure 2b), or spots are absent (Figure 2a). Penis in lateral view short robust and evenly curved (Figure 7a). Parameres evenly curved and tapering to apex (Figure 7g)	*Rhantus manjakatompo*

## Discussion

On Madagascar, *Rhantus* is a genus of the highland plateau. Like the *Rhantus* diversity in southeast Asia, Indonesia and Melanesia (Balke 2001), the genus is lacking from the lowland tropics in Madagascar. The central highland plateau of Madagascar (about 40% of the island) however, is almost completely degraded and very little of the original forests remains. Manjakatompo forestry station is one of three small forest relics remaining of the high plateau together with Ambohitantely Special Reserve and the Anjozorobe Forest. In November of 2011 and January 2012 we carried out fieldwork in Manjakatompo forestry station, investigating the aquatic beetle fauna. Although we have sampled aquatic beetles across Madagascar at hundreds of localities, nowhere else was the *Rhantus* fauna richer both in individuals and species than in Manjakatompo. All three endemic species of Madagascar existed here in healthy populations, two of which are only known from one other locality outside of Manjakatompo. Manjakatompo is clearly a small and fragile but important forest refugium which *Rhantus* and other highland fauna may depend on.

Manjakatompo is located in the province of Antananarivo, region Vakinakaratra and district of Ambatolampy, at 17 km to the west of the city Ambatolampy (19°22'S, 47°16'E). It lies on the eastern slope of the Ankaratra mountain massif of Quaternary volcanic origin. The altitude ranges between 1550 and 2643 m with the highest peak, Tsiafajavona, being the third highest on Madagascar. Forests are humid and the climate follows a pattern of cold and dry austral winter and a warm and wet austral summer (annual rainfall around 2000mm) (Vences et al 2002). The average temperature of the coldest month is 5–10°C, but can drop below zero at higher altitudes. The station covers an area of 8320 ha, with only 650 ha of natural forest and 2300 ha replanted with exotic trees (Goodman et al. 1996). Even the natural forest portion is composed of largely secondary forest mixed with exotic trees. Manjakatompo forestry station was established in 1923 to preserve the relic of primary forests that remained at the time (Andriampenitra 2007).

The forest relics have been kept partly thanks to legal protection more or less effectively exerted by the agents of the Forestry Station, with its status as Integral Reserve (Andriampenitra 2007). However, no part of the Ankaratra massif is part of the protected area network of reserves and national parks with a higher level of protection. According to our personal observation, and local information, the Manjakatompo forest is constantly exposed to serious incidents such as commercial operation, fire and slash and burn agriculture, so called “tavy”. In 2008, about 70 ha of the forest surface was burnt (Martin 2008). Inventories of the herpetofauna in Manjakatompo also found a specialised montane fauna with some 10–15% being endemics of the Ankaratra massif (Vences et al. 2002). Vences et al. (2002) were concerned that changes like increased use of pesticide, increased cattle (zebu) grazing, or aquaculture and release of fish, could cause a serious threat to the montane fauna. All these three factors are also known causes by which the aquatic insect fauna can drastically change, leading to the replacement of endemics by widespread opportunists. Kremen et al. (2008) used distribution data of various organism groups to model and optimize where additional protected areas on the island would come best to use for conserving additional components of the endemic fauna and flora not already under protection. The Ankaratra massif was part of the proposed new areas. While the flora and fauna show similarities to the Andringitra massif, which we can confirm based on the *Rhantus* fauna, it also has unique components and characteristics (Vences et al. 2002, Goodman et al. 1996), and remains a highly prioritized area for increased protection.

## Supplementary Material

XML Treatment for Rhantus manjakatompo

XML Treatment for Rhantus bouvieri

XML Treatment for Rhantus latus

## Appendix

Supplementary table include detailed information of all examined specimens. (doi: 10.3897/zookeys.350.6127.app) File format: Microsoft Excel file (xls).
